# The utility of psychometric network analysis for the study of social isolation, loneliness, and social support in persons with cancer: methodology and future directions

**DOI:** 10.3389/fragi.2026.1799545

**Published:** 2026-04-02

**Authors:** Thomas V. Merluzzi, Andrea Chirico, Tommaso Palombi, Errol J. Philip

**Affiliations:** 1 Department of Psychology, University of Notre Dame, Notre Dame, IN, United States; 2 Department of Developmental and Social Psychology, Sapienza, University of Rome, Rome, Italy; 3 Department of Dynamic and Clinical Psychology, and Health Studies, Sapienza, University of Rome, Rome, Italy; 4 School of Medicine, University of California, Los Angeles, CA, United States

**Keywords:** cancer, loneliness, network analysis, social isolation, social support

## Abstract

It has been well-established that social isolation, loneliness, and inadequate social support are critical determinants of psychological and physical outcomes in persons with cancer. In contrast to latent variable analysis, upon which most current research is based, psychometric network analysis offers a framework for understanding how these social factors relate to specific symptoms, coping resources, and wellbeing in the context of cancer. Although very few studies in this area of research have used network analysis methods, this presentation focuses on a small number of recent network-analytic studies in oncology and other related examples, emphasizing methodological choices, stability procedures, interpretation of centrality indicators, and global network analyses. In addition, recommendations are presented for future research, such as longitudinal models, network analysis of interventions, and multifaceted data integration that might inform translational work in psycho-oncology. Psychometric network analysis holds strong potential to advance psychosocial oncology through the identification of specific modifiable targets that might help mitigate social isolation, loneliness, and inadequate support.

## 1 Introduction

There is long-standing evidence that social isolation, loneliness, and inadequate social support have significant detrimental health effects, marked by increased morbidity in serious illnesses and mortality (Nelson and Faquin, 2024; Sherman et al., 2024; Kraav et al., 2021; Cacioppo and Hawkley, 2003). While social isolation (i.e., small or no social network), loneliness (i.e., the undesirable feeling of being alone and/or not close to others), and inadequate social support (i.e., not having others to rely on when you need them) may be distinct to some extent, they converge in their relationship with cancer morbidity and mortality (Liu et al., 2025; Cheng et al., 2025; Zhao et al., 2024). Evidence shows known correlates of social isolation, such as perceived stress, negative emotional states, elevated blood pressure, immune system impairment, and elevated stress hormones (Infurna et al., 2025; Cacioppo and Hawkley, 2003), that contribute to poorer quality of life and disease outcomes.

Social isolation in persons with cancer is a complex scenario that includes some antecedent causes such as sociodemographic factors (e.g., race, income, and age), concurrent factors such as psychological distress and negative coping styles, health-related factors such as non-cancer comorbidities (Wheldon et al., 2025; Wang et al., 2024; Merluzzi et al., 2021), and developmental changes in the perception of one’s health (Merluzzi and Nairn, 1999). Moreover, evidence also indicates that cancer and its treatment can affect social functioning (Liang et al., 2022; Haviland et al., 2017), leading to poor adherence to treatment regimens, social disengagement (Kenen et al., 2006), and isolation (Liang et al., 2022), which can increase emotional distress and physical symptoms (Sherman et al., 2024; Eisenberger and Lieberman, 2004).

This brief synopsis of the detrimental effects of social isolation, loneliness, and inadequate social support provides a backdrop for the focus on network analysis to study these social variables. For the most part, psychometric network theory conceptualizes items on measures as components of a system that can be visually displayed (Borsboom et al., 2021). Thus, for example, items referred to as “nodes” on a depression inventory (Bickel et al., 2024) are the components of a system, with partial correlations between pairs of nodes, referred to as “edges.” Thus, network analysis may provide a deeper insight into the structure of psychopathology, illness-related symptoms, and social factors at the item level (Borsboom et al., 2021).

A brief primer in network methodology is presented, followed by a review of a select group of network-analytic studies. Given that, to date, very few network analysis studies have focused on social isolation, loneliness, and social support in cancer, the review emphasizes methodological approaches and future directions.

## 2 Brief synopsis of psychometric network analysis

The information presented on network analysis is drawn extensively from Briganti et al. (2024) and Borsboom et al. (2021). In psychometric network analysis, a partial correlation network is constructed in which an edge represents the partial correlation between two nodes (items) after controlling for all other nodes in the network. The most common network estimation technique is the Gaussian graphical model (GGM) for continuous data. An important next step is the use of the graphical least absolute shrinkage and selection operator (GLASSO), which prevents spurious connections by shrinking small correlations to zero, thereby creating a network that is more stable, parsimonious, and interpretable. The Ising method is used for estimating network structures based on binary variables. In the graphic representation of data, items from separate inventories (e.g., depression and loneliness) or lists (e.g., physical and psychological symptoms) are color-coded to distinguish different communities of nodes. The edge width represents the strength of the partial correlations (e.g., thicker edges for stronger connections). Color can also be used to indicate the direction of relationships (e.g., green for positive and red for negative). Thus, the estimated network is visualized in graphic form, with nodes representing variables and edges representing their partial correlations.

There are several metrics that describe the importance of nodes in the network. Strength centrality is the sum of all the edges (i.e., partial correlations) for a node, without respect to the sign. Nodes with high strength centrality are important because changes (e.g., over time) in these nodes can have a significant impact on the entire network. Betweenness centrality is a measure of the degree to which a certain node lies on the shortest path between two other nodes, representing an important connector. While betweenness centrality is the standard way to quantify a node’s “bridge-ness,” “bridge centrality” sometimes also refers to a node’s role in connecting different communities of nodes. Nodes with high betweenness centrality are critical for the flow of information because they function to connect other nodes in the network. Those nodes may also be important because of their theoretical or clinical relevance, such as being factors in feedback loops that sustain psychopathology. Thus, betweenness or bridge centrality metrics have the potential for identifying mechanisms for change. The variants of strength and bridge centrality are strength EI and bridge EI (EI = expected influence). For bridge EI, the weighted edges of a node are summed by taking the sign of the edges into account for nodes that are directly connected to a target node (one-step EI) or one or two edges from a target (two-step EI). Strength EI is the sum of all the edges of a node, with the sign taken into account.

The correlation of the stability (CS) coefficient is a metric that assesses the robustness and reliability of centrality indices or edge weights. The CS coefficient is calculated using a case-dropping bootstrap method, which involves repeatedly computing the network measure (e.g., strength centrality) on various-sized random subsets of the data to determine its stability with smaller subsets. A CS value of 0.75 would indicate that a centrality measure would not vary significantly from the original value when including only 25% of the edge weights. CS values should be greater than 0.50 to be unambiguously interpreted.

Nodes with high centrality have important implications because they may inform the subsequent development of more precise assessment tools and interventions. That is, by developing interventions that target highly central nodes, one may create a ripple effect throughout the entire network, potentially alleviating other symptoms simultaneously. Likewise, a “coping” node with high bridge centrality (e.g., seeking support) between “loneliness” (i.e., a distress node) and “satisfaction with life” (i.e., a quality-of-life node) may help identify a potential mechanism for change.

There are several metrics that apply to the network as a whole. The global density is the ratio of the number of reliable edges present in the network to the total number of possible edges between all nodes; higher levels of density indicate more tightly connected networks where nodes are strongly linked. The global density can also be statistically compared between networks. Another overall metric, global strength invariance, compares the overall level of connectivity between two or more networks by examining the sum of the absolute values of all edge weights. A significant difference in global strength indicates that one network is more or less interconnected overall compared to another. In cases of significant differences at the overall level, specific weighted differences between node pairs can be compared statistically between networks.

The network comparison test (NCT) is used to compare the overall structural differences between networks. NCT is computed on differences in invariance measures between two networks (network structure invariance, global strength invariance, edge invariance, and invariance of centrality measures). These global measures can be used with cross-sectional data to test differences between groups or with longitudinal data to test changes over time or as a function of an intervention. Other approaches to longitudinal network analysis include the graphical vector autoregressive (GVAR) model, a regression-based model in which each node at a time *t+1, t+2, … t + n* is regressed on nodes at time *t*. Thus, the edges in this model, called directed edges, temporally predict the effects across time. Multilevel VAR (mlVAR) extends the GVAR to test both within-person temporal networks and between-person network changes/differences. That is, this model computes temporal effects as deviations from the individuals’ within-person averages, thereby capturing intra-individual effects around the means (Speyer et al., 2021) and the differences between networks (e.g., treatment *versus* control groups). Finally, centrality measures alone could be used to test the differences between networks (McNally, 2016).

## 3 Overview of network studies

While network analysis is gaining momentum in psycho-oncology, there is a paucity of studies that focus on loneliness, social isolation, and social support. Taking this into consideration, in-depth reviews and critiques have been included for a few studies that have focused on these topics, primarily emphasizing methodological issues, future research, and clinical applications. In addition, a study that did not focus on cancer but was exemplary in methodological innovation was included, which might advance network and intervention research for persons with cancer. The review of those studies concludes with recommendations for a network analysis research agenda that would have implications for intervention development.

### 3.1 Hybrid model of latent profile analysis and network analysis

Yang et al. (2025) used a hybrid model of latent profile and network analysis to contrast networks between two distinct groups. Their design included a cross-sectional study of 256 patients with colorectal cancer who completed established measures of loneliness, social anxiety, and social avoidance. Incorporating those measures into a latent profile analysis, they used fit statistics to identify two subgroups (LSI: low social isolation and HSI: high social isolation). The network data included the social variables used to establish the groups, along with measures (scale scores, not items) hypothesized to correlate with social isolation, namely, symptom burden, family functioning, internet use, and religious beliefs. In the LSI network, loneliness was a very important node (i.e., a very high strength centrality index), with very strong edges with social anxiety and social avoidance and a strong edge between loneliness and symptom burden but no other significant edges. In the HSI network, loneliness was likewise important (moderately-high-strength centrality index), with very strong edges with family functioning and symptom burden and much weaker edges with social anxiety and social avoidance. Thus, family functioning (HSI reported significantly more dysfunction than LSI) and loneliness formed the core of the HSI network. Generally, the two networks were not very complex, although the HSI network was somewhat more complex. While global strength was not different between the groups, the authors did not test global density, which appears to be higher in the HSI group’s network. There were several interesting potential differences in centrality measures between the two groups, with centrality scores appearing to diverge (e.g., loneliness, religious beliefs, and family functioning); however, these differences were not tested statistically or discussed in detail. In summary, because interpretive tools such as differences in centrality measures and network density were not used and the difference in the centrality index of religious beliefs was not discussed, the interpretability of the results was limited, as was the utility of this study in guiding the design of interventions.

### 3.2 Network analysis of risk and resources

Schellekens et al. (2020) and Chirico et al. (2024) tested risk and resources models (i.e., stress and coping) in persons with cancer, and these studies are included to highlight theory- and/or hypothesis-driven (Morse et al., 2021; Merluzzi et al., 2019a) network research. Schellekens et al. (2020) compiled scale scores from self-report measures (N = 384 cancer patients) to establish risk nodes (physical symptoms, social withdrawal, and helplessness) and protective factors (acceptance of illness, perceived benefit, disengagement from unattainable goals, and reengagement in new goals), along with individual depression items. While social withdrawal was not identified as a central node, the network analysis suggested that depression nodes (mood, loss of enjoyment, and worthlessness) and acceptance of illness had the highest strength centrality values, indicating that they could be potential targets for intervention. However, the most significant edges in the network followed a literal, visual throughline of edges from risk to resources (physical symptoms–fatigue–helplessness–acceptance–benefit finding), with implications for intervention development. Bridge EI centrality (two-step) of acceptance of illness may have highlighted this tightly knit chain of nodes further by taking into account nodes one and two edges away from the acceptance node, which may indicate a focus on intervention. In this study, the use of scale scores limited the degree of fine-grained analysis of individual items; however, it allowed for the inclusion of many constructs, establishing the basis for follow-up work that could provide a detailed analysis of the risk- and resilience-related item nodes. Overall, this study demonstrated that network analysis can be theory-driven and lead to implications for developing interventions.

In another cross-sectional study with a large sample (N = 992 mixed diagnosis cancer patients), Chirico et al. (2024) began with a latent variable model based on an abridged (24-item) version of the Distress Screening Schedule (Philip, 2012). The authors conducted a confirmatory factor analysis (CFA) that included two factors representing risk (depression and anxiety) and four factors representing resource factors (receiving social support, seeking support, agentic coping, and satisfaction with medical care). The analysis of items (as opposed to scale scores) allowed for a comparison of the fit of the CFA model with the network solution. Importantly, the network replicated the CFA structure of the DSS; both showed acceptable fit, with the network representing a slightly better fit.

The depression item *felt lonely/isolated* was the centrality node with the highest strength, and a coping efficacy resource item (*found ways of dealing with my illness*) had the second highest strength. In addition, *seeking support, perceiving one’s doctor as caring*, and *receiving support* also had high strength centrality, indicating that social resource indicators are potential targets for interventions aimed at reducing loneliness. Finally, the strongest edges connecting different nodes were between *seeking support* and *receiving emotional support* and between *coping efficacy* and *seeking support*, indicating an agentic perspective on seeking and obtaining social support that might form the basis for an intervention to counter loneliness (Merluzzi et al., 2019b). The large sample in this study provided greater reliability and stability in all the network parameters and the integration of latent and network models.

Taken together, these two studies demonstrate how network analysis can be linked to latent variables and, in the case of Chirico et al. (2024) can delve beyond scores on measures to items representing specific symptoms of disorders and resources, which may be translated into tailored treatments.

### 3.3 Network analysis of mediation and mechanisms

Network analysis can also add depth to typical model testing, such as mediation, with both cross-sectional and longitudinal designs. The study reviewed in this section was not conducted in a cancer setting, but it represents an excellent and innovative network project in terms of methodology that can serve as a springboard for future cancer research. Luo et al. (2025) hypothesized that rumination is the mechanism (mediator) that accounts for the relationship between loneliness and depression. That is, rumination is an example of preservative cognition that can prolong the stress response and hypervigilance (Luo et al., 2025). As detailed by Chirico et al. (2024), Luo et al. (2025) first tested a latent variable mediation model (loneliness→ rumination→ depression) and found significant direct and indirect effects, confirming the preservative cognition hypothesis. The items from the measures used to conduct the mediation analysis (UCLA 3-item Loneliness Scale, Ruminative Responses Scale (RRS), and Patient Health Questionnaire-9) were included in the network analysis. Interestingly, bridge EI (one-step, i.e., taking the edge sign into account) outperformed bridge centrality with respect to CS-coefficients. Among the loneliness and rumination communities of the nodes, the highest bridge EI nodes, namely, *how often do you feel alone* and *how often do you think about how alone you feel,* firmly connected loneliness with rumination. Then, when considering loneliness and depression nodes, *how often do you feel isolated from others* and *feeling down, depressed, and hopeless* had the highest bridge EI values compared to all the other nodes. Thus, for both the latent variable and network models, the mediation of rumination supported the preservation cognition hypothesis. Moreover, focusing on decreasing ruminative behavior, bridge EI symptoms may “deactivate” the cycle between loneliness and depression. This study is an excellent example of how network analysis can be theory-driven, utilize a multimethod approach to test the theory, and guide the development of targeted interventions.

## 4 Conclusions and future directions for network analysis of loneliness, social isolation, and inadequate social support in the context of cancer

Research utilizing network analysis of loneliness, social isolation, and social support is in its early stages, and the few studies reviewed were significantly heterogeneous, thereby limiting both synthesis across studies and broad-scale inferences. Moreover, caution is needed going forward because these social constructs may have different causes, meanings, and manifestations in the cancer setting (Schellekens et al., 2020; Hartung et al., 2019). As a parallel example, Hartung et al. (2019) found that depressive symptoms were more frequent but less strongly intercorrelated in cancer patients than in controls; thus, social aspects of depression (i.e., loneliness) may not manifest in the same way as in the general population. Thus, further research on the nuances and meanings of loneliness, social isolation, and inadequate social support in the context of cancer is required (Marconi et al., 2024) to elucidate how such constructs may be meaningfully different in persons with cancer than in the general population. In this respect, network analysis may be particularly helpful in identifying specific prominent nodes and configurations of nodes that differ between loneliness, social isolation, and inadequate social support, thereby adding nuance to their characterization and potentially indicating focal points for developing interventions based on these network differences.

Future network analyses should also focus on changes during cancer treatment (Shao et al., 2025) or interventions for emotional problems and loneliness. It would be helpful to identify controllable central nodes (e.g., seeking support) that could form the basis of interventions and to understand how emotional, physical, and social variables relate to one another, along with how stable or changeable those relationships are over time and across treatment (Kalantari et al., 2022). This approach may add precision to existing programs that focus on mitigating loneliness and isolation (Perissinotto et al., 2019) and explain their relationship with somatic/physical and psychological symptoms (Doppenberg-Smit et al., 2025; Kalantari et al., 2022). The combination of social variables, physical and psychological symptoms, and quality-of-life outcomes (Kim L. et al., 2025; Kim Y. et al., 2025) in network analyses may present a more holistic picture of traversing cancer and take into account varying kinds of social “pain” that might adversely affect the course of cancer care and survivorship (Eisenberger and Lieberman, 2004) and lay the groundwork for ways to remedy it.
